# Subependymal Giant Cell Astrocytoma Apoplexy: A Case Report and Systematic Review

**DOI:** 10.7759/cureus.34784

**Published:** 2023-02-08

**Authors:** Alaa N Turkistani, Mahmoud Fallatah, Aliaa H Ghoneim, Fahad Alghamdi, Saleh S Baeesa

**Affiliations:** 1 Neurological Surgery, King Faisal Specialist Hospital and Research Centre, Jeddah, SAU; 2 Surgery, King Saud Bin Abdulaziz University for Health Sciences College of Medicine, Jeddah, SAU; 3 Radiology, King Abdulaziz University Faculty of Medicine, Jeddah, SAU; 4 Pathology, King Abdulaziz University Faculty of Medicine, Jeddah, SAU; 5 Neurosciences, King Faisal Specialist Hospital and Research Centre, Jeddah, SAU; 6 Neurological Surgery, King Abdulaziz University Faculty of Medicine, Jeddah, SAU

**Keywords:** subependymal giant cell astrocytoma, tuberous sclerosis, hemorrhage, apoplexy, terson syndrome

## Abstract

Subependymal giant cell astrocytoma (SEGA) is the most common intracranial tumor in tuberous sclerosis (TS) patients. The tumor generally localizes in the proximity of Monro's foramen; as it grows, it subsequently causes hydrocephalus and increases intracranial pressure (ICP). However, acute symptoms of increased ICP due to intratumoral bleeding rarely manifest in SEGA patients.

We present a 27-year-old male with TS who presented due to hemorrhagic complications of SEGA with intratumoral bleeding and vitreous orbital hemorrhage. We then conducted a systematic review with four databases (PubMed, Web of Science, Google Scholar, and Cochrane) to identify similar cases using the following keywords: "Subependymal giant cell astrocytoma," "Hemorrhage," "Haemorrhage," and "Bleeding."

Our review identified 12 articles reporting 14 cases of hemorrhagic complications of SEGA in addition to our case report. The median age of diagnosis was 21 (range 5-79) years with unequal gender distribution (M:F ratio, 11:4). Headache was the most presented symptom, followed by hemiparesis, seizure, altered mental status, visual deterioration, and headache accompanied by seizure. TS was seen in most of the cases (80%). Gross total resection (GTR) was achieved in 53.5% of the patients. Regarding the clinical outcome, 66.7% had a good outcome, 20% died, and 13.3% had no report of their outcomes. No tumor recurrence was seen in the cases with a reported duration of follow-up.

Catastrophic presentation of SEGA apoplexy is a rare occurrence. We present a case report with a systematic review and discuss SEGA apoplexy's possible pathophysiology and outcome.

## Introduction

Tuberous sclerosis (TS) is an inherited autosomal-dominant neurocutaneous disorder characterized by tumors involving many organs, including the brain, heart, kidneys, and skin [1]. A clinical scoring system was developed that divides the diagnostic criteria for TS into major and minor features. Subependymal giant cell ependymoma (SEGA) is a significant feature and the most common intracranial tumor in patients with TS [2]. The prevalence rate of SEGA in patients with TS ranges from 5% to 20%. In addition, solitary SEGAs in the absence of TS-related lesions have been reported; these resulted from somatic mosaicism of the TSC gene or de novo mutations at the TS locus [3]. 

The updated World Health Organization (WHO) classification of central nervous system tumors considers SEGA a grade 1 tumor, and it is generally localized in proximity to the foramen of Monro [4]. SEGA is typically a slow-growing tumor that causes blockage of the cerebrospinal fluid pathway, leading to progressive symptoms due to hydrocephalus and increased intracranial pressure (ICP); however, catastrophic presentation due to intratumoral bleeding is a rare occurrence. 

We describe a 27-year-old male previously diagnosed with TS who presented with rapid consciousness and visual acuity deterioration due to intratumoral hemorrhage in SEGA with simultaneous intraocular hemorrhage. Moreover, we conducted a systematic review of the literature regarding intratumoral hemorrhagic complications (apoplexy) of SEGA and summarized their management and outcome.

## Case presentation

A 27-year-old male patient presented to our emergency department with a history of progressive visual deterioration over two days associated with a mild headache. There is no history of seizures, and the patient has a positive family history of TS. He had butterfly distribution of facial freckling and small hypopigmented macules on examination. His Glasgow coma score (GCS) was 15/15, and there were no motor-focal neurological deficits in the extremities. Ophthalmologic examination revealed bilateral papilledema with vitreous and retinal hemorrhage suggesting Terson syndrome. Visual acuity was poor, with light perception in the right eye and 20/300 in the left eye.

**Figure 1:**
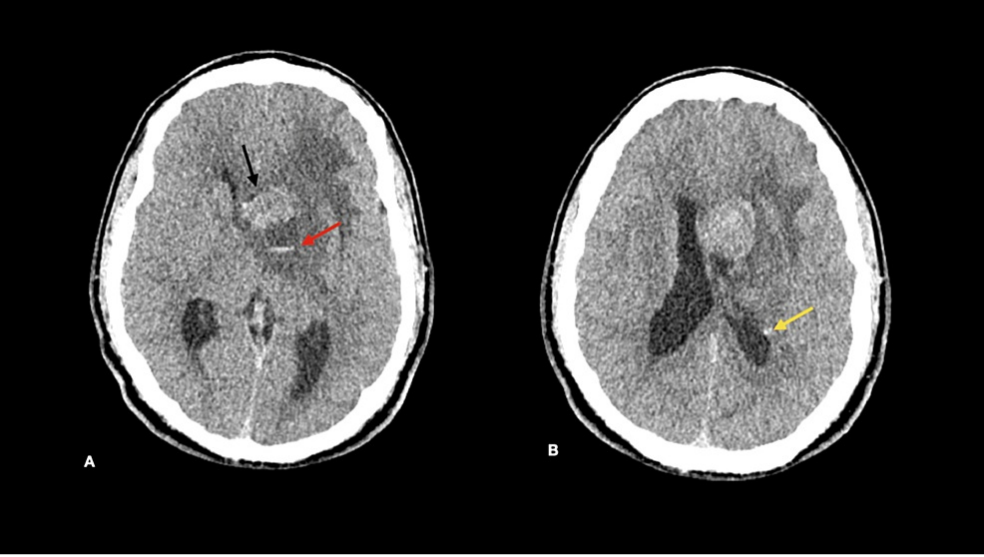
Axial unenhanced CT brain. A. showing the subependymal heterogeneously hyperdense mass (black arrow) with the cystic area showing acute blood-fluid level (red arrow). B. showing calcified subependymal nodule (yellow arrow).

An emergency unenhanced computed tomography (CT) scan of the brain was performed. It showed a well-defined mass involving the subependymal surface and projecting into the frontal horn of the left lateral ventricle. It is heterogeneous but mainly hyperdense, with fluid density areas and blood-fluid levels indicating hemorrhages (Figure 1A). The mass has surrounding parenchymal vasogenic edema, causing a mass effect on the interventricular foramen, resulting in the right lateral ventricle dilatation. A few calcified subependymal nodules are also seen (Figure 1B).

**Figure 2:**
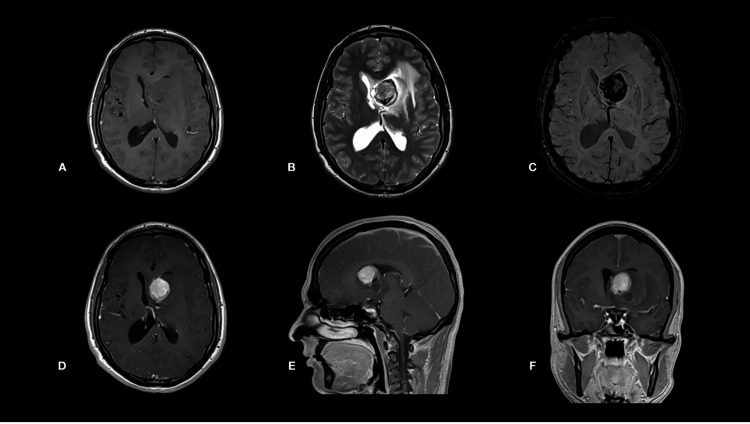
Preoperative brain MRI scan revealed (A) axial T1- and (B) T2-WI scans showing the intermediate signal intensity subependymal mass with dark T2 signal foci and cystic region. There is a significant mass blooming effect in the SDI-WI scan (C), indicating hemorrhages. C. Axial, sagittal, and coronal T1-WI scans (D-F) following intravenous contrast administration revealed heterogeneous enhancement of the left lateral ventricular tumor.

Brain magnetic resonance imaging (MRI) showed left intraventricular mass with solid and cystic components measuring 27 X 25 mm at maximum diameter. The solid component showed intermediate T1 and T2 signal intensity with different ages of subacute hemorrhage, and there is a significant blooming on the susceptibility-weighted imaging (Figure 2A-2C). The cystic region showed a blood-fluid level with a focal high T1 signal indicating acute hemorrhage. The tumor has heterogenous enhancement on the post-contrast T1-sequence and leads to (Figure 2D-2F). The subependymal nodules showed intermediate signal intensity on the T2-weighted imaging. Additionally, there were multiple subcortical high signal intensities in keeping with tubers and linear radial bands extending from the ventricles to the brain surface (Figure 2B).

**Figure 3:**
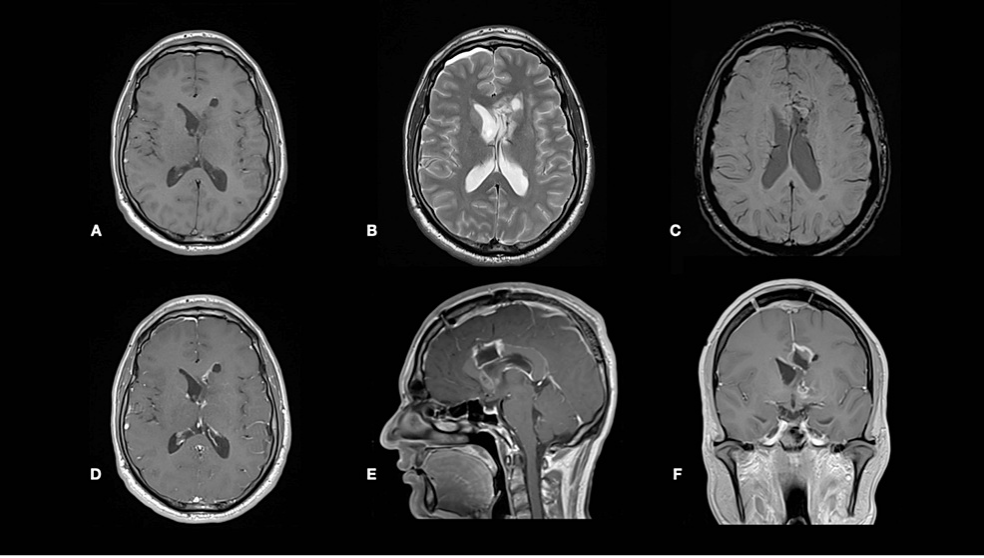
Postoperative brain MRI scan (A-F) after right frontal craniotomy and transcallosal microsurgical resection of the tumor revealed complete resection and resolution of surrounding hemorrhagic changes.

The patient underwent left frontal craniotomy, and complete resection of a hemorrhagic lateral ventricular tumor was achieved through a transcallosal approach. He had an uneventful postoperative recovery and was discharged home after a week with stable vision. His early postoperative MRI scan revealed complete resection of an intraventricular tumor and resolution of peritumoral ischemic changes (Figure 3).

**Figure 4:**
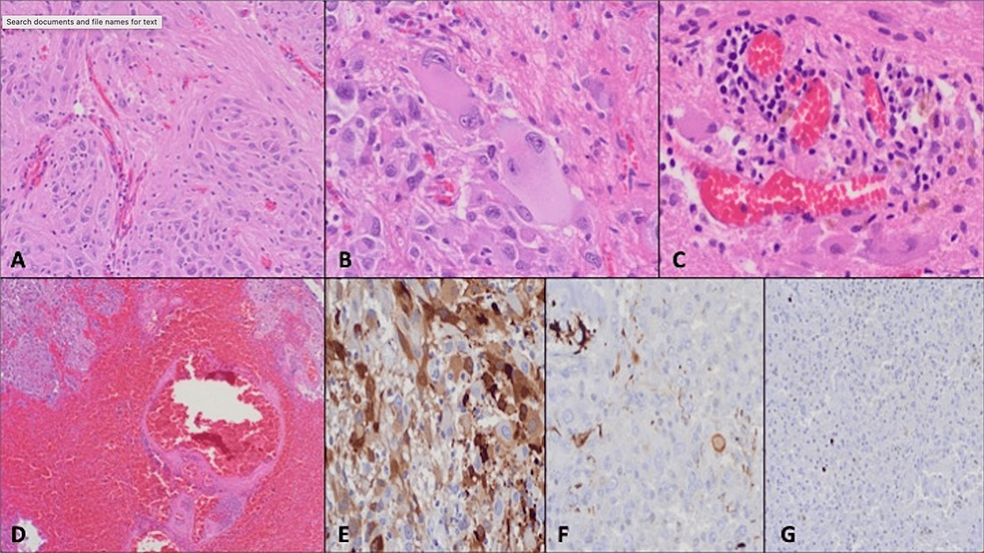
Histopathological examination of the resected intraventricular tumor confirming the diagnosis of SEGA. A. Large gemistocytic-like cells admixed with elongated spindle cells arranged into sweeping fascicles with a variable fibrillary background. B. Giant cells with a ganglionic appearance and multinuclei. C. Foci of inflammatory cells, including lymphocytes and mast cells, within a rich vascular stroma. D. Wide area of hemorrhage within and adjacent to the tumor (left corner). E & F. Variable immunoreactivity for NSE and GFAP, respectively. G. Ki-67 proliferation index: Very low. SEGA: subependymal giant cell astrocytoma, NSE: neuron-specific enolase, GFAP: glial fibrillary acidic protein

Pathologic examination of the resected lesion revealed a well-circumscribed neoplasm composed of large, plump, gemistocytes-like cells admixed with smaller, elongated, and spindle cells, arranged into sweeping fascicles with variable fibrillary background (Figure 4A). A few giant cells with a ganglionic appearance and single or more nuclei were also identified (Figure 4B). Foci of inflammatory cells, including lymphocytes and mast cells, were seen within a rich vascular stroma (Figure 4C). Apart from that, large areas of hemorrhage within and adjacent to the tumor (Figure 4D). The tumor cells demonstrated variable immunoreactivity for the glial fibrillary acidic protein gene (GFAP), neuron-specific enolase (NSE), and CD56 (Figures 4E, 4F). IDH-1 was negative in the tumor cells. The tumor cells showed no mitosis, and the Ki-67 proliferation index was very low (Figure 4G).

The patient had a satisfactory neurological recovery and was discharged home in good condition. He maintained good neurological, cognitive, and motor function during follow-up; however, he had a slight visual acuity improvement. The follow-up MRI scan at one year showed no tumor recurrence.

## Discussion

Systematic review and analysis

Study Objectives

The study's primary objective was to review all reported cases of hemorrhagic SEGA and compare the clinical outcome between patients harboring hemorrhagic SEGA with TS and those pertaining to hemorrhagic SEGA without TS.

Search Methods

We performed a systematic review searching PubMed, Web of Science, Google Scholar, and Cochrane databases using a combination of the following keywords with Boolean operators (OR/AND): “Subependymal giant cell astrocytoma,” “Hemorrhage,” “Bleeding.” It was conducted based on Preferred Reporting Items for Systematic Reviews and Meta-Analyses (PRISMA) guidelines [5]. Additionally, candidate articles from the reference list of the eligible studies were also reviewed. 

Inclusion and Exclusion Criteria

Intratumoral hemorrhagic SEGA was first reported by Waga et al. in 1977 [6]; however, we couldn’t find an electronic copy of the paper, and thus it was not included in our study. Therefore, we included all published studies which reported at least one case of hemorrhagic SEGA after 1977 until January 12, 2022. We only included studies that confirmed SEGA by histopathological examinations. Diagnosis of intratumoral hemorrhage by radiological images or explicit mention by authors was considered acceptable. The presence of TS was considered acceptable by fulfilling the established clinical criteria or the reported presence of a genetic mutation. Studies conducted in a non-English language, as well as autopsy diagnoses, were excluded.

Selection of Studies

The eligibility of each article found through the database search was assessed and determined by two independent reviewers (AT and MF) by screening the title/abstract and then reviewing the full-text versions of the articles. All disagreements were resolved either by a consensus or by arbitration of a third reviewer (SB).

Data Collection

Extracted data from each eligible article included: the number of hemorrhagic SEGA patients, demographic data, clinical presentation, the presence of TS, type of surgery, tumor recurrence, clinical outcome, and the duration of follow-up.

Quality Assessment

The quality and risk of bias assessment of included studies were appraised by two independent reviewers (AT and MF). In addition, the quality of case reports was appraised using the Joanna Briggs Institute Critical Appraisal Checklist for Case Reports, which consists of eight yes/no/unclear questions (Table 1).

**Table 1:** Joanna Briggs Institute (JBI) Critical Appraisal Checklist for Case Reports

JBI checklist questions	Kalina P, et al. (1995) [14]	Kim MS, et al (2003) [16]	Stavrinou P, et al. (2008) [17]	Sterman H, et al. (2012) [18]	Wadhwa R, et al. (2011) [19]	Ogiwara and Morota (2013) [20]	Kim JY, et al. (2017) [21]	Movahed-Ezazi M, et al. (2020) [8]	Barbiero FJ, et al. (2021) [9]
Were patient’s demographic characteristics clearly described?	Yes	Yes	Yes	Yes	Yes	Yes	Yes	Yes	Yes
Was the patient’s history clearly described and presented as a timeline?	Yes	Yes	Yes	Yes	Yes	Yes	Yes	Yes	Yes
Was the current clinical condition of the patient on presentation clearly described?	Yes	Yes	Yes	Yes	Yes	Yes	Yes	Yes	Yes
Were diagnostic tests or assessment methods and the results clearly described?	Yes	Yes	Yes	Yes	Yes	Yes	Yes	Yes	Yes
Was the intervention(s) or treatment procedure(s) clearly described?	No	Yes	Yes	Yes	Yes	Yes	Yes	Yes	Yes
Was the post-intervention clinical condition clearly described?	No	No	Yes	Yes	Yes	Yes	Yes	No	Yes
Were adverse events (harms) or unanticipated events identified and described?	Yes	Yes	Yes	Yes	Yes	Yes	Yes	Yes	Yes
Does the case report provide takeaway lessons?	Yes	Yes	Yes	Yes	Yes	Yes	Yes	Yes	Yes

The quality of case series was appraised by Joanna Briggs Institute Critical Appraisal Checklist for Case Series, which consists of 10 yes/no/unclear questions [7]. A high risk of bias was considered if a study scored "Yes" less than 49% of the quality criteria, moderate risk of bias if it scored "Yes" between 50% and 74% of the quality criteria, and low risk of bias if it scored "Yes" in at least 75% of the quality criteria. A consensus resolved all disagreements (Table 2).

**Table 2:** Joanna Briggs Institute (JBI) Critical Appraisal Checklist for Case Series

JBI checklist questions	Shepherd CW, et al. (1991) [12]	Sinson G, et al. (1994) [13]	Kim SK, et al. (2001) [15]	Giordano F, et al. (2019) [22]
Were there clear criteria for inclusion in the case series?	Yes	Yes	No	Yes
Was the condition measured in a standard, reliable way for all participants included in the case series?	Yes	Yes	Yes	Yes
Were valid methods used for identification of the condition for all participants included in the case series?	Yes	Yes	Yes	Yes
Did the case series have consecutive inclusion of participants?	Yes	Yes	No	Yes
Did the case series have complete inclusion of participants?	Yes	Yes	No	Yes
Was there clear reporting of the demographics of the participants in the study?	No	Yes	Yes	Yes
Was there clear reporting of clinical information of the participant?	Yes	Yes	Yes	Yes
Were the outcomes or follow up results of cases clearly reported?	Yes	Yes	Yes	Yes
Was there clear reporting of the presenting site(s)/clinic(s) demographic information?	Yes	Yes	Yes	Yes
Was statistical analysis appropriate?	Yes	Yes	Yes	Yes

Systematic Review Results

**Figure 5:**
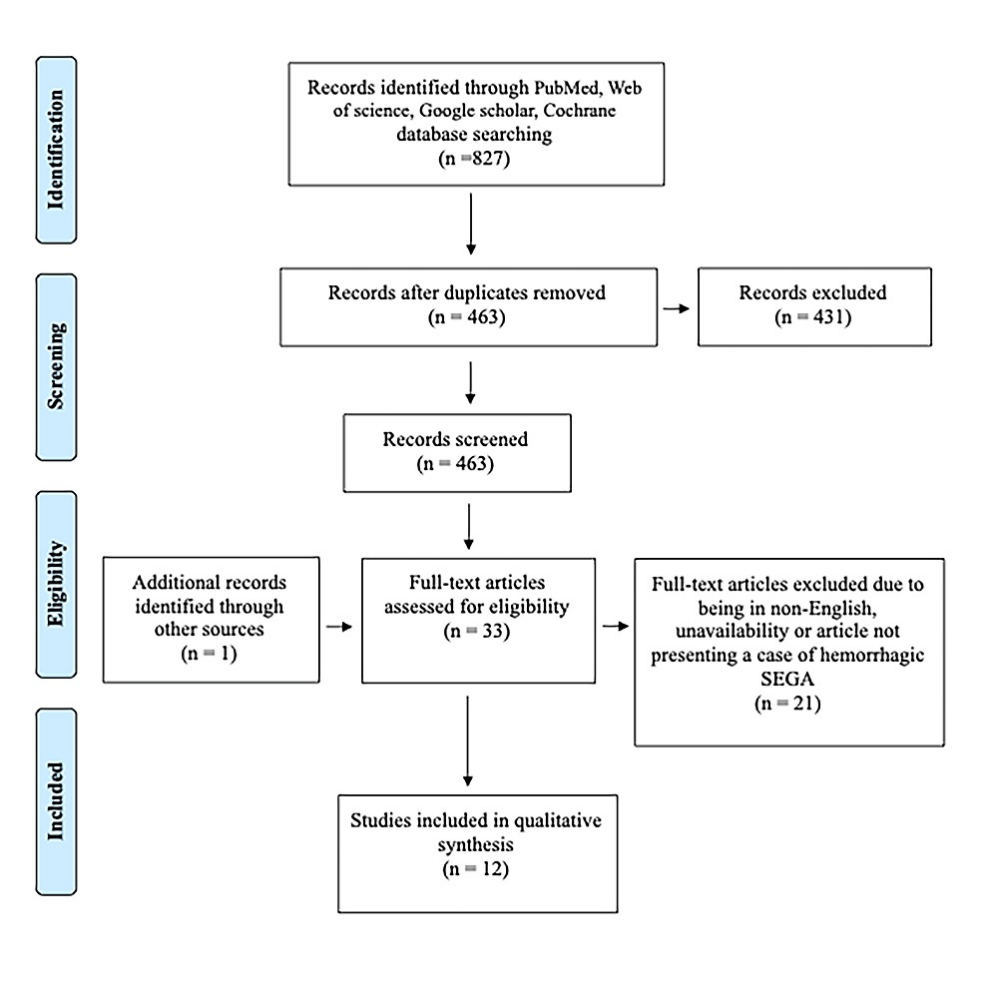
Flowchart diagram of our search mechanism in accordance with the Preferred Reporting Items for Systematic Reviews and Meta-Analyses (PRISMA).

A total of 827 studies were identified from the selected databases, and 364 were removed as duplicates. Among the remaining 463 studies, 431 were excluded after title/abstract screening, and 21 were excluded after reviewing the full text. Twelve articles that fulfilled the inclusion criteria were included in the study (Figure 5). 

Demographic Data

A total of 15 cases with hemorrhagic SEGA have been identified in the literature, including our patient, summarized in Table 3. The median age of diagnosis was 21 (range 5-79) years with significant male-to-female predominance, 11 (73.3%) and four (26.7%), respectively. TS was associated with hemorrhagic SEGA in 12 (80%) patients, and three (20%) have no features of TS.

**Table 3:** Summary of reported subependymal giant cell astrocytoma (SEGA) with apoplexy cases in the literature TS: tuberous sclerosis, STR: subtotal resection, GTR: gross total resection, AMS: altered mental status, NA: not applicable, NS: not specified

Author (year) (reference)	Age (years) /Sex	TS	Presentation	Surgery	Recurrence	Outcome	follow up
Shepherd et al. (1991) [12]	31/ M	Yes	NS	NS	NS	Death	NS
Sinson et al. (1994) [13]	21/M	Yes	Headache, lethargy	STR	NS	death	NS
Kalina et al. (1995) [14]	17/ F	Yes	Seizure, lethargy	Tumor resection (NS extent of resection)	NS	NA	NS
Kim et al. (2001) [15]	9/ M	Yes	Headache	STR	None	Good	14 months
Kim, Min-Su, et al (2003) [16]	12/M	No	Headache, Seizure	GTR	None	NS	3 months
Stavrinou et al. (2008) [17]	33/ M	No	Headache	GTR	NS	Good	NS
H. Sterman (2012) [18]	8/ M	Yes	Headache, vomiting	GTR	None	Good	22 months
Rishi Wadhwa (2011) [19]	50/ M	Yes	Headache, vomiting	GTR	NS	Good	NS
Ogiwara and Morota (2013) [20]	5/ M	Yes	Headache, vomiting	GTR	None	Good	6 months
	21/ M	Ye s	Hemiparesis, vomiting lethargy	STR	None	Good	3 years
Kim JY (2017) [21]	10/ F	No	Hemiparesis, vomiting lethargy	GTR	None	Good	2 years
Flavio Giordano et al. (2019) [22]	34/F	Yes	Epilepsy	GTR	NS	Death	NS
Movahed-Ezazi M et al. (2020) [8]	79/F	Yes	Lethargy, AMS, decreased oral intake	STR	NS	Good	NS
Barbiero FJ, et al (2021) [9]	21/M	Yes	Headache	Tumor resection	NS	Good	NS
Current case (2022)	27/ M	yes	Headache, Visual deterioration	GTR	No	Good	2 years

Clinical Presentations

Most of the cases presented with headache (seven, 46.7%), followed by hemiparesis (two, 12.5%), seizure (two, 12.5%), altered mental status (one, 6.7%), visual deterioration (one, 6.7%), and headache accompanied by seizure (one, 6.7%).

Imaging and Pathological Characteristics

Non-contrast CT scan of the reported cases demonstrated hemorrhagic hyperdense lesions constantly arising adjacent to the foramen of Monro, mainly in the lateral ventricle, causing obstructive hydrocephalus. The reported measures of the mass ranged from 1.5 cm to 5.6 cm. MRI scans performed in nine cases typically show a well-defined cystic, solid mass with mixed intensities in T1 and T2 weighted images with heterogeneous post-contrast enhancement. Moreover, multiple subependymal nodules and subcortical calcification were observed in four cases associated with TS.

Histopathologically, all cases showed the typical features of SEGA, namely, neoplastic astrocytes in glial fibrillary background, large cell gemistocytes-like with rounded nuclei intermingled with spindle cells, and multinucleated giant cells, prominent vascularity as well as areas of hemorrhages, some inflammatory cells such as mast cells, positive immunohistochemistry for GFAP, low Ki-67 labeling index and negative IDH-1. In addition, genetic analysis was performed recently in two cases both showed TSC1 germline mutation [8,9].

Operative Characteristics and Outcome

Gross total tumor resection was achieved in eight (53.5%) cases, subtotal resection in four (26.7%) and three (20%) cases; the extent of the tumor resection was not explicitly mentioned. The clinical outcome was good after surgical treatment in 10 cases, while death occurred in three cases, all of which had TS. One patient was due to an intramural hemorrhage, and the other two were due to treatment complications. In two cases, the outcome was not reported. Of those with a good outcome, tumor recurrence after resection was not seen during the follow-period in seven patients, while for the rest, their duration of follow-up was not reported (Table 3).

SEGA and tuberous sclerosis

SEGA is a benign, slowly growing tumor classified as WHO grade I that is frequently seen in patients with TS with 0.5% to 20% of patients [1,4,10]. Patients who present primarily with SEGA only rarely manifest the classic triad of adenoma sebaceum, seizure, and mental retardation, which characterizes tuberous sclerosis commonly diagnosed in childhood. SEGAs are typically located near the foramen of Monro. Because their location and growth potential can cause increased intracranial pressure, obstructive hydrocephalus, focal neurologic deficits, and death [11]. Acute symptoms can develop insidiously by progressive growth of the tumor and abruptly by intratumoral bleeding [8,9,12-22]. 

Diagnostic Criteria

The preoperative diagnosis of SEGA considers the patient's age and clinical condition and the tumor's location. In most cases, TS is diagnosed according to the clinical diagnostic criteria, of which the presence of SEGA is the most significant [23]. Besides, a sporadic presentation is relatively common, with approximately 85% of the cases resulting from new mutations (therefore, no familial history of TS is usually obtained), making the preoperative diagnosis extremely challenging.

Genetic Analysis

The tumor suppressor genes TSC1 on chromosome 9q34 and TSC2 on chromosome 16p13 encode the tuberin and hamartin, respectively. Mutations in the TSC1/2 genes are not observed in 20% of patients diagnosed with TSC. The disease in patients without mutations is less severe than in those with TSC1/2 mutations [24]. It has been reported that solitary SEGAs that do not show any clinical evidence of TS can result from somatic mosaicism [11]. Other reports have described solitary SEGAs with isolated somatic TSC2 mutations or amplification of exons on the TSC1 gene. Solitary cases of SEGA without mutation may be due to epigenetic alteration in tuberin or hamartin [25]. Our case demonstrated atypical facial freckling, which favors the diagnosis of TS, and the brain imaging results and family history; a genetic study was not performed. 

Pathophysiology of SEGA Hemorrhagic Presentation

The overall incidence of acute intracranial hemorrhage in brain tumors is 4.6% and 13.2 % in children [18]. Secondary metastatic tumors are more prone to intracerebral hemorrhage than primary tumors, and glioma is more prone to bleed among all primary lesions. About 3-7% of gliomas are associated with hemorrhage, and less than 1% in low-grade gliomas [26]. Despite their benign nature, intratumoral bleeding has been reported in low grades gliomas, and the prognosis in some cases is unfortunate. It is thought that the mechanism of tumoral bleeding associated with malignant tumors is related to vascular invasion, structural abnormalities, or tumor outgrowing its blood supply, causing necrosis and hemorrhage, which will lead to fragile blood vessels wall and increases the susceptibly of hemorrhagic events [27]. Some studies have demonstrated that an over-expression of vascular endothelial growth factor (VEGF) in brain tumor cells or metastatic brain tumors [28] leads to tumor-associated intratumoral hemorrhaging. Additionally, matrix metalloproteinases (MMP) overexpression might play a role in metastatic brain tumor hemorrhage. Nevertheless, low-grade lesions (grades I and II) do not present with either one of these features, making it difficult to explain the bleeding in these tumors [29]. 

The hemorrhage of SEGA has rarely been reported in the literature, and only 14 cases were collected from the English literature on SEGA (Table 3). Most cases presented with headaches, two patients presented with hemiparesis, and two presented with seizures. The results are summarized in Table 1. Our patient's main complaint was decreased visual acuity, and ophthalmologic examination revealed bilateral papilledema with virtuous and retinal hemorrhage suggesting Terson syndrome. Some studies investigated the histopathological features of SEGA. Prominent vascularization and an angiocentric pattern, as well as perivascular pseudopalisading, were described [18,27,30]. A high degree of neovascularization was demonstrated by immunohistochemistry using CD-31 (a marker for vascular endothelium) [31]. However, there is no evidence that these findings increase the risk of hemorrhages. Despite the unknown mechanism of bleeding, SEGA apoplexy is vital and was associated with significant morbidity and mortality.

Management and Outcome of SEGA Apoplexy

Treatment of SEGA is essentially based on extensive surgical excision, which can be curative. However, the appropriate time for surgery is still controversial for small asymptomatic lesions [25,32]. Although early intervention to those small lesions decreases the operative morbidity compared to large lesions, the surgery carries a risk of complications. As intratumoral bleeding is a rare presentation of brain tumors in general and in SEGA precisely [33], the decision of surgical resection should be individualized. Most cases with intratumoral bleeding reported in the literature underwent surgical resection in acute deterioration with intratumoral hemorrhage to reduce the mass effect caused by the lesion. The outcome was stable in the last reported cases compared to earlier reported cases. This could be related to advancement in the field, early intervention, and understanding of the behavior of intratumoral hemorrhage. The current case had urgent surgical gross total resection of the lesion with external ventricular drain (EVD) insertion. The patient did very well postoperatively, EVD was removed after a few days, and the patient was discharged home in stable condition. 

Recent studies showed a reduction of SEGA tumors using radiosurgery or the mechanistic target of rapamycin (mTOR) inhibitors. Even though the sporadic cases limited the role of radiosurgery in SEGAs, these reported results suggest radiosurgery may be an additional option for SEGAs where complete resection has not been safely achieved [34,35]. In addition, the reduction of SEGA using mTOR inhibitors has also been reported [36]. Yet, the response is temporary, lasting only as long as the medication is used. In addition, its toxicity may exceed its benefits due to the necessity for long-term use of these medications.

## Conclusions

SEGA apoplexy is a rare and catastrophic presentation that may occur in any age group. Our systematic review and case illustration recommend urgent surgical excision of the tumor and associated hematoma as the best treatment with a good outcome. The pathophysiological mechanism of intratumoral bleeding of SEGA is still unknown. Future genetic analysis is required to understand the behaviors of SEGA and the possible predisposing factors for hemorrhagic complications.
